# Underwater Pile Foundation Defect Detection Method Based on Diffusion Probabilistic Model and Improved PointMLP

**DOI:** 10.3390/s25185639

**Published:** 2025-09-10

**Authors:** Tongyuan Ji, Dingwen Zhang

**Affiliations:** 1 School of Transportation, Southeast University, Nanjing 211189, China; zhang@seu.edu.cn; 2 China Design Group Co., Ltd., Nanjing 210014, China

**Keywords:** point cloud, diffusion probabilistic model, PointMLP, attention mechanism, pile foundation defect detection

## Abstract

To detect damage in underwater pile foundations, we propose a new method based on the diffusion probability model and improved PointMLP. First, PCA-ICP registration is carried out for the point cloud data from different stations using a sonar system. A variety of filtering algorithms and the Random Sample Consensus (RANSAC) method are employed to obtain a complete point cloud of the pile foundation. The pile foundation defect point cloud is generated and enhanced based on the diffusion probability model. The feature attention mechanism is added to the PointMLP, and then the improved PointMLP is trained to identify the defect of the pile foundation. In our study, the point cloud of a wharf pile foundation was collected, and the experimental results effectively identified the damage to the pile foundation. Up to 95% accuracy was achieved for the calculated volume. The volume error of the damage was 0.0756 m^3^, with an accuracy of 95.238%. Thus, this method can provide technical support for detecting underwater pile foundation defects and avoiding the occurrence of major accidents.

## 1. Introduction

In recent years, with the continuous development of sonar equipment, the acquisition of underwater point cloud data has become more convenient, and its quality and cost performance are continuously improving. It is widely used in the three-dimensional modeling of large scenes, underwater target identification and detection, and underwater pile foundation detection during wading projects [1,2,3,4,5,6,7,8]. In the latter, a sonar is installed on the two-degrees-of-freedom head, and the scanning is driven by motor rotation. However, the three-dimensional point cloud data obtained for the underwater pile foundation and surrounding terrain are incomplete and cannot fully capture the state of the pile foundation. Xu [9] et al. proposed a method for detecting defects in underwater pile foundations using binocular vision and the YOLOV8 neural network model for recognition. However, this imaging method is ineffective at capturing the three-dimensional characteristics of underwater pile foundations. As point cloud data intrinsically contain three-dimensional features, it is necessary to perform scanning at multiple locations and register the findings to obtain a complete set of three-dimensional point cloud data.

The accurate registration of point cloud data normally uses the initial value provided by the rudimentary scanning of point cloud data from two different angles. Then, the iterative operation is carried out according to the initial value before the transformation matrix is solved. The iterative closest point algorithm (ICP) is the basis of iterative registration proposed by Besl and McKay [10]. It directly utilizes all point cloud information to carry out an iterative transformation. This algorithm has high accuracy and is often used for precise registration. Most iteration methods have been improved based on this approach. Liu et al. [11] proposed an improved principal component analysis (PCA)-based fast ICP matching algorithm. By solving the principal component of two sets of point clouds, the respective PCA coordinate system is formed. Then, the K-D tree is used to quickly search for the nearest point to improve the traditional ICP method and registration efficiency.

There is a scarcity of point cloud data for pile foundation defects, so overfitting problems often occur in the training of the model, and for defect detection, an extreme imbalance between positive and negative samples usually exists. To solve the above problems, point cloud generation should be carried out according to the original point cloud data. Goodfellow et al. [12] proposed a generative deep learning model called Generative Adversarial Network (GAN). This network has a simple structure and can perform unsupervised learning on the main features of original datasets; however, it is difficult to train, and the model is prone to collapse. To avoid complexity during training, researchers from Cornell University and NVIDIA proposed a three-dimensional (3D) point cloud generation model, PointFlow [13], based on a flow model, which generated 3D point clouds by modeling them as distributions. Luo et al. [14] proposed a diffusion probability model using noise distribution to replace the original distribution. By transforming the noise distribution into a desired shape, it enabled point cloud data generation equivalent to the process of reverse diffusion.

Point cloud classification is a method that classifies the point cloud into different point clusters, where the same point cluster has similar or the same attributes. Since the convolutional neural network (CNN) has achieved great success in image segmentation, classification, and other fields, researchers have gradually applied CNN to point cloud processing. However, point clouds are disordered, so many researchers first conduct regularization preprocessing for point clouds, convert point cloud data into regular data-like images, and then use the traditional CNN method [15]. The multi-view method adopts the above ideas to transform 3D point cloud data into a two-dimensional plane from multiple angles, which is then projected onto a picture. Then, the projected image convolves in two dimensions [16,17,18]. However, the transition from 3D to 2D loses a large amount of information, and the original point cloud data cannot be accurately classified. The PointNet [19] network presented a new idea, which abandoned the regularization of point cloud data and directly convolved the input of point cloud coordinates. It independently learned each of the cloud points and extracted global features for classification. PointNet++ [20] and PointMLP [21] have achieved higher accuracy in point cloud classification and segmentation based on the improvement of PointNet. With the significant progress of Transformer in natural language and image processing, Guo [22] and Zhao [23] et al. designed a point cloud processing neural network based on Transformer, which also achieved good accuracy. To improve the efficiency and accuracy of pile defect detection, an attention mechanism was introduced based on PointMLP to allow focus on the information most critical to the current task and reduce the attention given to other input information.

This article proposes a method based on PCA-ICP combined with an improved PointMLP point cloud segmentation network. By scanning the point cloud data of underwater pile foundations, defects and normal states can be accurately classified. The contributions of this article are as follows:

(1) The PCA-ICP registration method, multiple filtering algorithms, and the Random Sample Consensus (RANSAC) method can be used to obtain the complete pile point cloud.
(2) An underwater pile defect detection method based on the diffusion probability model and improved PointMLP is proposed, along with a slice method to calculate the pile defect volume.

The rest of this article is organized as follows: Section 2 introduces the complete pile foundation point cloud according to the preprocessing method. Section 3 presents the diffusion probability model, which increases the number of data points, and the improved PointMLP with attention mechanism, which recognizes the defect type. In Section 4, the experiments are described to verify the defect detection method. The conclusions are detailed in Section 5.

## 2. Point Cloud Data Preprocessing Method

**Figure 1:**
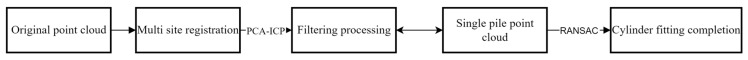
Preprocessing flowchart.

In this study, the sonar system was installed on a 2-DOF cradle head, and the cradle head was rotated by a motor to scan the underwater scene to obtain cloud data from the pile foundation’s mud surface and other views. The density of the point cloud was irregular, including a large amount of data, outliers, and noise, so the point cloud data obtained could not be directly used, and preprocessing was necessary. The preprocessing method is shown in Figure 1. The methodconsists of three stages: multi-site point cloud registration, point cloud filtering, and point cloud completion, as follows:

(1) Multi-site point cloud matching: The PCA-ICP registration algorithm is used to register scanning point clouds from different sites.
(2) Point cloud filtering: Voxel filtering, straight pass filtering, spherical area filtering, Gaussian statistical filtering, and radius filtering are used to obtain point clouds from individual pile foundations.
(3) Point cloud completion: The point cloud completion of the pile foundation is achieved using the RANSAC fitting cylinder.

PCA-ICP registration is used for point cloud data from different sonar sites, so the point cloud registration is performed from multiple perspectives.

(1) PCA method

Suppose the point set, ***P*** = {*P_i_*}, *i* = 1, 2, …, *n*, is three-dimensional data representing the point cloud distribution. Firstly, the mean value and covariance matrix of the point set are calculated using Equation (1), and the three feature vectors of the covariance matrix are represented as three vertical directions, respectively. The spatial Cartesian coordinate system of the point set is established using the XYZ coordinate axis, and the corresponding transformation parameters of the covariance matrix of the two-point cloud are obtained using the mean value as the origin of the coordinate system. The coordinate system of the point cloud matched with the coordinate system of the target point cloud is adjusted using the desired transformation parameters, and the pre-matching of the point cloud PCA is complete.

(1) $$\mathrm{COV}=\frac{1}{N}\sum_{i=1}^{N}\left(P_{i}-\bar{p}\right){\left(P_{i}-\bar{p}\right)}^{T}$$

(2) ICP method

The ICP method is the nearest point iteration method. It uses the nearest corresponding point iteration to accomplish the alignment and matching of multi-viewpoint clouds, which can be regarded as the least squares based on the spatial transformation optimal problem. The basic idea of this algorithm is to transform the corresponding points between two clouds into a three-dimensional matrix and convert the space coordinates of the point cloud to the reference point cloud space coordinate system to achieve point cloud registration.

First, PCA coarse registration is performed with a large overlap, and then ICP registration is performed based on coarse registration. In other words, the rudimentary PCA registration of point cloud *M* and point cloud *N* obtains a rotation and translation matrix after the first rotation and translation to obtain $M^{\prime }$ and *N*. Then, point clouds *P* and *Q* are approximately PCA-aligned after the first rotation and translation to obtain $P^{\prime }$ and *Q*. The second rotation and translation matrix is obtained using $M^{\prime }$ and *N* for ICP registration, and then the point clouds $P^{\prime }$ and Q are used for ICP registration. $P^{\prime \prime }$ and *Q* are obtained after the second rotation and translation.

Firstly, point clouds *P* and *Q* with overlapping parts are registered using PCA to obtain $P^{\prime }$ and *Q*. Overlapping areas between point clouds make it possible to carry out the ICP algorithm.

The basic principle of the ICP algorithm is as follows: in the target point cloud *N* and source point cloud $M^{\prime }$ to be matched, the nearest point (***M**_i_*, ***N**_i_*) is found according to certain constraints, and the optimal matching parameters ***R*** and ***T*** are calculated to minimize the error function *E* (***R***, ***T***), which is the following:

(2) $$E\left(R,T\right)=\frac{1}{n}\sum_{i=1}^{n}{\left\|M_{i}-(RN_{i}+T)\right\|}^{2}$$

where *n* is the number of nearest point pairs; ***N**_i_* is a point in the target point cloud *N*; ***M**_i_* is the nearest point in the source point cloud *M* corresponding to ***N****_i_*; ***R*** is the rotation matrix; and ***T*** is the translation vector.

After horizontal plane calibration, there are still spot clouds as well as multiple pile foundations and noise point clouds in the data, so it is necessary to use a filtering algorithm to obtain the point cloud data of a single pile foundation. In this study, the voxel filter was first used to compress the point cloud, reduce the number of points, and increase the subsequent filtering processing speed. Then, a pass-through filter was used to filter out the points with values below the threshold in the specified dimension. Subsequently, spherical region filtering was performed to obtain the points of the specified spherical region. Finally, point cloud radius filtering based on connectivity analysis was used to preserve the point cloud meeting assumptions, and the point cloud of a single pile foundation was obtained.

Since sonar scans are performed at the locations of measuring points, the point cloud of a single pile foundation obtained via filtering is incomplete. In this paper, the RANSAC method was used to fit the cylinder, the point cloud coordinates were converted into polar coordinates, and the unscanned angles of the pile foundation were calculated. Finally, the point cloud completion was carried out in the calculated angle area to obtain complete point cloud data for a single pile foundation.

## 3. Underwater Pile Foundation Defect Detection Methods

In this paper, we propose an underwater pile foundation defect detection method based on the diffusion probability model to produce a pile foundation defect dataset, and the PointMLP classification network is trained to obtain the final model. The preprocessed actual pile foundation point cloud data is then analyzed by the model for defect detection.

### 3.1. Diffusion Probability Model

**Figure 2:**
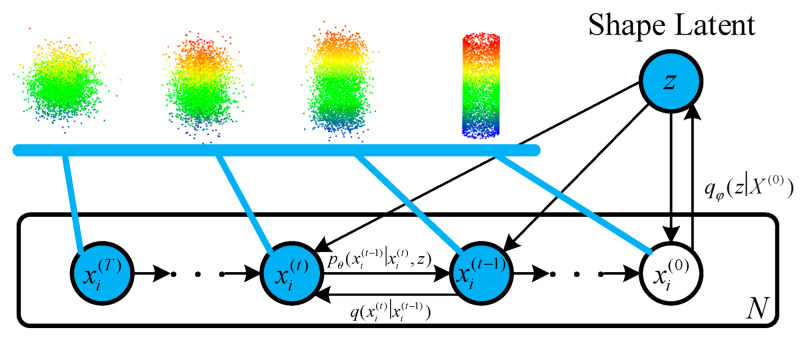
Diffusion probability model.

Inspired by the diffusion process in non-equilibrium thermodynamics, the points in a point cloud are compared to particles in a non-equilibrium thermodynamic system in contact with a heat bath. In the presence of a heat bath, the positions of the particles evolve randomly, diffusing from the original distribution to a noise distribution (entropy increase theory). The generation of a point cloud is, therefore, equivalent to learning the back-diffusion process, transforming the noise distribution into a distribution of the desired shape. The model is shown in Figure 2.

For the point cloud, $X^{\left(0\right)}={\left\{x_{i}^{\left(0\right)}\right\}}_{i=1}^{N}$ consisting of *N* points, each ***x****_i_* can be considered a point independently sampled from the data with the distribution $q(x_{i}^{\left(0\right)}\left|z\right.)$, where *z* is the shape’s latent coefficient. A diffusion model consists of two processes: the diffusion process and the reverse process.

The purpose of the diffusion process is to gradually map $X^{\left(0\right)}$ to a multidimensional normal distribution (Gaussian noise) via a Markov chain, i.e.,

(3) $$q\left(x_{i}^{\left(1:T\right)}\left|x_{i}^{\left(0\right)}\right.\right)=\prod_{t=1}^{T}q\left(x_{i}^{t}\left|x_{i}^{t-1}\right.\right)$$

where $q\left(x_{i}^{t}\left|x_{i}^{t-1}\right.\right)$ is the Markov diffusion kernel, defined as the Gaussian distribution $N(x^{\left(t\right)};\sqrt{1-\beta_{t}}x^{t-1},\beta_{t}I)$. The variance-scheduling hyperparameter *β_t_* controls the diffusivity of the process. The process corresponds to the iterative addition of small amounts of Gaussian noise, which eventually transforms the target into a multidimensional normal distribution that is independent in different dimensions $x_{i}^{\left(T\right)}$.

Different from the forward diffusion process, which only adds noise to the points, the reverse process is generated by sampling based on a normal distribution, aiming to recover the desired shape from the input noise:

(4) $$p_{\theta }(x^{\left(0:T\right)}\left|z\right.)=p(x^{\left(T\right)})\prod_{t=1}^{T}p_{\theta }(x^{\left(t-1\right)}\left|x^{\left(t\right)},z\right.)$$

where $p_{\theta }(x^{\left(t-1\right)}\left|x^{\left(t\right)},z\right.)$ is defined as $N(x^{\left(t-1\right)}\left|\mu_{\theta }(x^{\left(t\right)},t,z\right.),\beta_{t}I)$, and ***µ****_θ_* is the estimated mean value learned by a neural network implemented with parameter *θ*. Through this process, we can gradually eliminate Gaussian noise, pass a set of points sampled from the starting distribution of the standard normal distribution, $p(x_{i}^{\left(T\right)})$, through a reversed Markov chain to obtain a point cloud with the target shape, and finally generate the data that matches the target distribution.

In this study, the initial dataset was obtained, which was then used to train a diffusion probability model to generate an expanded dataset of pile foundation point cloud data in preparation for training a PointMLP network.

### 3.2. Improving the PointMLP Network Model

PointMLP is a deep learning architecture specially designed for point cloud data processing. Its core purpose is to extract and enhance the geometric characteristics of point clouds through a multilevel perceptron (MLP) and affine transformation, and optimize the training efficiency of deep networks using residual connections. The architecture mainly consists of two key modules: the geometric affine module and the ResP Block.

The function of the geometric affine module is to carry out an affine transformation on input point cloud data to enhance the expression of geometric features. This module typically includes MLP and Batch Normalization (BN) layers and introduces nonlinearity in conjunction with the ReLU activation function to ensure that the model can learn more complex point cloud distribution patterns. The feature after the affine transformation can better adapt to the geometric changes (such as rotation, translation, or scaling) in the point cloud, improving the robustness of the model.

ResP Block is the core computing unit of PointMLP, which adopts the design of residual connection to relieve the gradient loss problem of the deep network. Each ResP Block contains multiple layers of MLP and BN internally and introduces a variety of feature operations such as Subtraction, Product, Hadamard Product, and Summation to enhance feature interaction. These operations enable the model to capture the local and global geometrical relationships of the point cloud in a more detailed way. In addition, the residual connection allows the gradient to be directly returned, ensuring efficient training of the deep network.

The entire architecture gradually extracts high-level features by stacking multiple ResP Blocks and finally outputs a point cloud representation with strong discrimination. The advantage of PointMLP lies in its simple and efficient MLP-based design, which avoids complex convolution or graph operations. Meanwhile, it ensures the expression and training stability of the model through geometric affine and residual mechanisms, making it highly effective in point cloud classification, segmentation, and other tasks.

**Figure 3:**
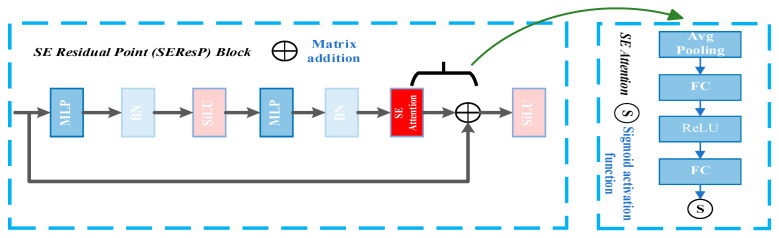
SEResP Block.

**Figure 4:**
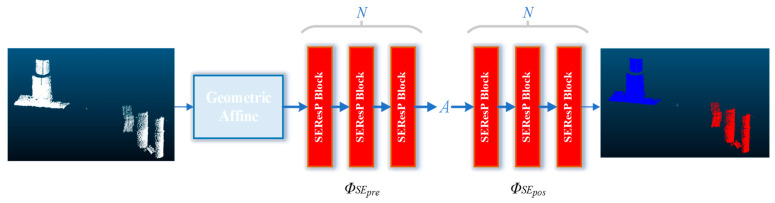
Attentional PointMLP structure.

The attention mechanism ensures the network knows which position to focus on, allowing it to automatically pay attention to important features, improving the network’s performance. To improve the efficiency and accuracy of the classification, this study added an attentional mechanism based on PointMLP. The attentional mechanism is shown in Figure 3. The original Rectified Linear Unit (ReLU) activation function was replaced with the Sigmoid Linear Unit (SiLU) function, which offers several advantages in deep learning architectures. This modification significantly enhances the network’s performance by introducing smooth, non-monotonic characteristics that help maintain stable gradients during backpropagation. When integrated with the Squeeze-and-Excitation (SE) attention mechanism, this combination ensures a robust feature extraction framework. The SE attention mechanism adaptively recalibrates channel-wise feature responses, while SiLU activation provides smoother decision boundaries and better gradient flow throughout the network. This synergistic effect results in improved feature representation and more stable training dynamics. The enhanced attentional PointMLP network architecture, as illustrated in Figure 4, demonstrates superior performance in processing point cloud data by optimizing the combination of these advanced components. Such architectural improvements lead to better convergence properties and more discriminative feature learning capabilities, particularly in complex 3D vision tasks where point cloud processing is crucial.

In this study, the dataset generated via the diffusion probability model was divided into the training set and the test set according to a ratio of 9:1, and the SE-attention PointMLP network was used for training. Finally, the classification network model was obtained. The point cloud data of a single pile foundation was fed into the classification network model, and identification and judgment were carried out to detect the existence and types of defects in the pile foundation.

### 3.3. Slice Method for Volume Calculation

A defective pile foundation is a type of irregularity. This study utilized a slice calculation method for the point cloud volume. The basic idea is to slice the point cloud along the *Z*-axis, then calculate the slice area and obtain the total volume by summing the sliced volumes. The slicing method consists of four stages: pile foundation point cloud slicing, contour boundary point finding and sequencing, slice area calculation, and point cloud volume calculation. The process is as follows:

Pile foundation point cloud slicing: between the minimum value and maximum value of the point cloud *Z*-axis, a fixed width is set to cut the point clouds from bottom to top and obtain point cloud slices successively.

Contour boundary point finding and sorting: the boundary point cloud is extracted using the grid division method, and then the out-of-order point cloud is sorted using the polar coordinate method.

Area calculation for slices: the area is calculated using the contour point area statistics method for the sorted contour points.

Point cloud volume calculation: the product of the area of each slice and the fixed width is accumulated to obtain the volume of the entire pile base point cloud. The key stages are the contour boundary point finding and sorting, and the slice area calculation.

#### 3.3.1. Contour Boundary Point Finding and Sorting

The gridding method is divided into three steps: (1) gridding; (2) finding the boundary grid; and (3) extracting the boundary lines. The first step is to create a minimum bounding box for the data point set and partition it with a rectangular grid at a specific interval. Then, the boundary grids are found and connected to form a “coarse boundary” consisting of boundary grids. Finally, for each boundary grid, it is determined whether the points within are boundary points according to certain rules. The initial boundary is connected and smoothed.

As the contour boundary points found are disordered and have a large impact on the subsequent area calculation, this study proposes using a polar coordinate method. The center of mass of the contour boundary points is calculated first, and then the polar coordinates of each point are calculated and sorted according to the polar coordinate angle.

#### 3.3.2. Slice Area Calculation

If n vertices $p_{0},\;p_{1},\;\ldots ,\;p_{n}$ are specified to form a polygon with the first and last vertices joined counterclockwise, the area enclosed can be calculated as follows:

(5) $$Area=\frac{1}{2}\left|\sum_{i=0}^{n-1}\left(x_{i}y_{i+1}-x_{i+1}y_{i}\right)\right|$$

where $x_{i},\;y_{i}$ are the coordinates of the vertices $p_{i}(i=0,\;1,\;\ldots ,\;n)$ for the outer contour polygon P of the sliced plane point cloud; i is the vertex number for the outer contour boundary polygon of the point cloud slice; and n is the number of point cloud slices.

In this study, the contour point area statistics method described above was used for the calculation of point cloud slice areas. The simplicity of the calculating process ensures the accuracy of the point cloud volume.

## 4. Experiments and Analysis

There are many different types of pile foundations, with various construction technologies. The quality of the concrete, mud, and other materials is uneven and, coupled with complex formation changes, pile foundations are prone to apparent damage. The local diameter of the pile foundation is less than or greater than the designed size. This not only affects the bearing capacity and endurance of the pile foundation and the stability of the superstructure, but also causes a vast amount of concrete waste. Aiming to discern the apparent damage of pile foundations, a pile foundation point cloud was used to test the pile foundation defect detection method proposed in this paper.

### 4.1. Point Cloud Registration

**Figure 5:**
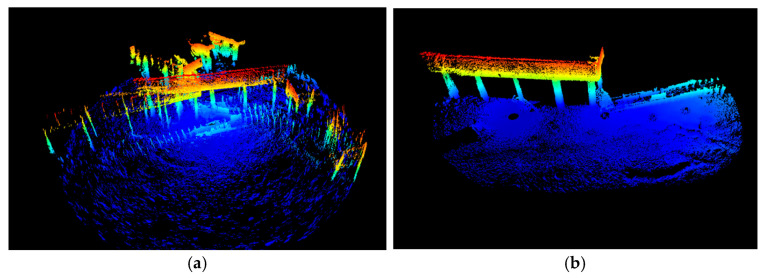
Registration results. (**a**) Pre-registration point cloud data; (**b**) point cloud data after registration.

The PCA-ICP algorithm was used to register the point cloud data collected using sonar from multiple perspectives, and the registration results are shown in Figure 5. The registration results of multiple pile foundation parts in the point cloud model are accurate.

### 4.2. Point Cloud Filtering and Point Cloud Complementation

**Figure 6:**
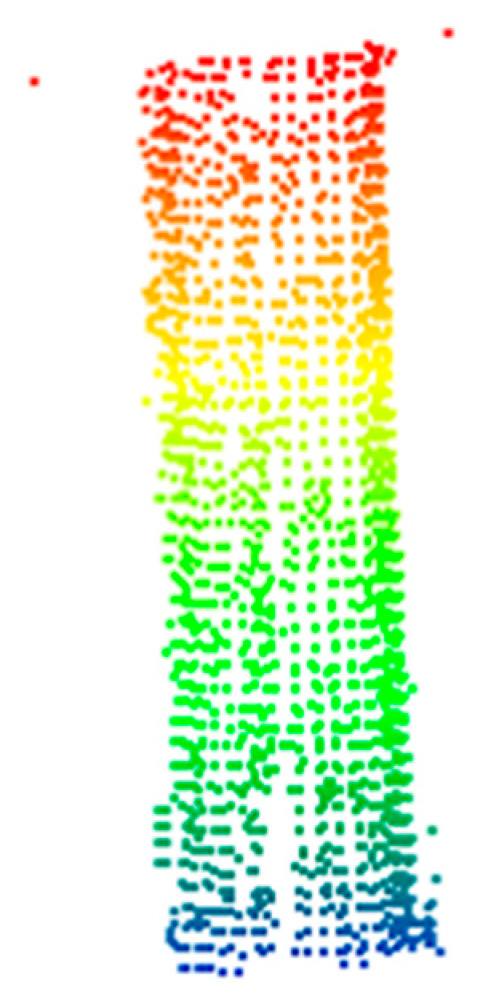
The result after filtering the pile point cloud.

**Figure 7:**
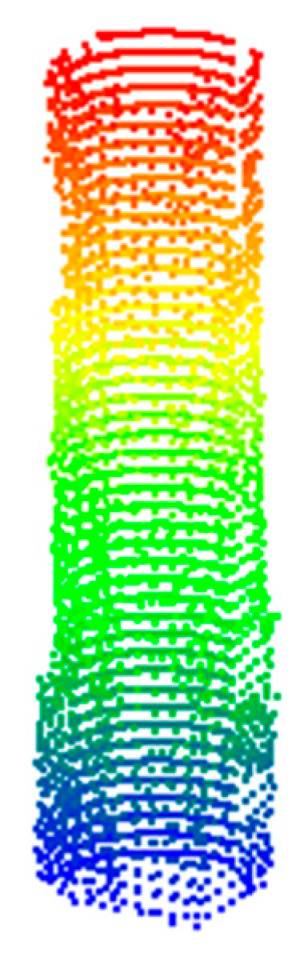
The result after completing the point cloud of the pile.

In the process of point cloud data processing, calibrated data include the point clouds of field scenes, multiple pile foundations, and noise. Therefore, it is necessary to separate single pile foundations from noise through filtering. The process begins with preliminary filtering of the calibrated point cloud, followed by spherical and radius filtering to eliminate interference points. Finally, voxel grid filtering is employed for downsampling. As shown in Figure 6, the point cloud data of a single pile foundation was successfully extracted through filtering. However, due to missing scans, the filtered point cloud data was incomplete. To address this, the RANSAC algorithm was first applied for cylinder fitting to reconstruct the pile foundation model. Then, the Cartesian coordinates of the selected point cloud were converted to polar coordinates to identify the unscanned angle regions, resulting in a complete set of pile foundation point cloud data, as shown in Figure 7.

### 4.3. Production of Pile Damage Datasets

**Figure 8:**
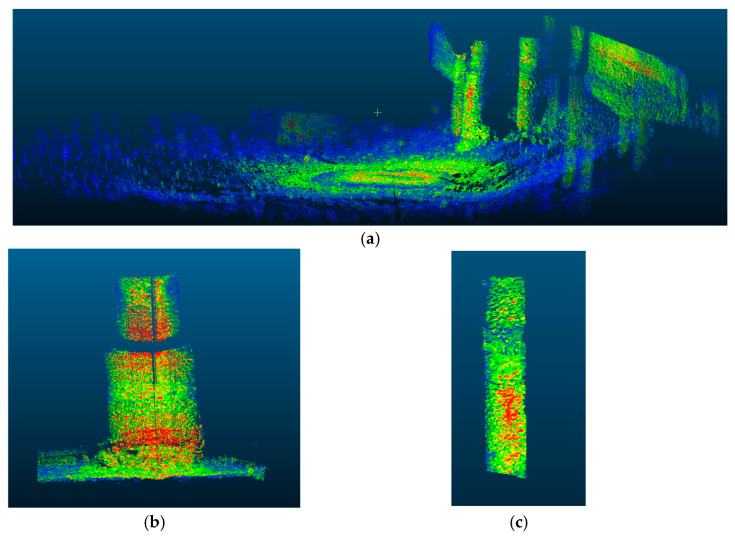
Training results. (**a**) Real point cloud; (**b**) defect point cloud; and (**c**) normal point cloud.

The complete point cloud data are shown in Figure 8a. There are five samples indicating defects from the point cloud data and 78 normal samples, as shown in Figure 8b,c. To address the problem of the small sample size of pile foundation point cloud data, this study produced a pile foundation defect dataset based on the diffusion probability model. Thus, 25 defect data points were obtained through data augmentation.

### 4.4. Detection of Pile Foundation Defects

The original PointMLP network and the improved attentional PointMLP network were trained separately using the pile damage dataset obtained in Section 4.3, and the results of 50 epochs of training are shown in Figure 8. SiLU and ReLU activation functions in the improved PointMLP network were compared, and the results are shown in Table 1. With the same model size of 15 MB, SiLU outperformed ReLU in terms of accuracy.

**Figure 9:**
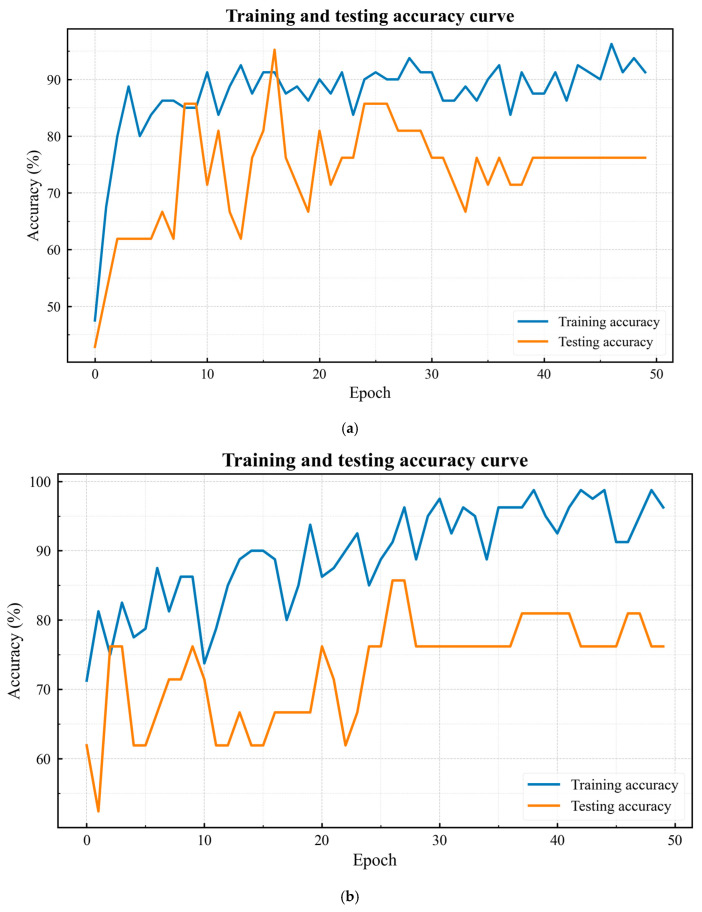

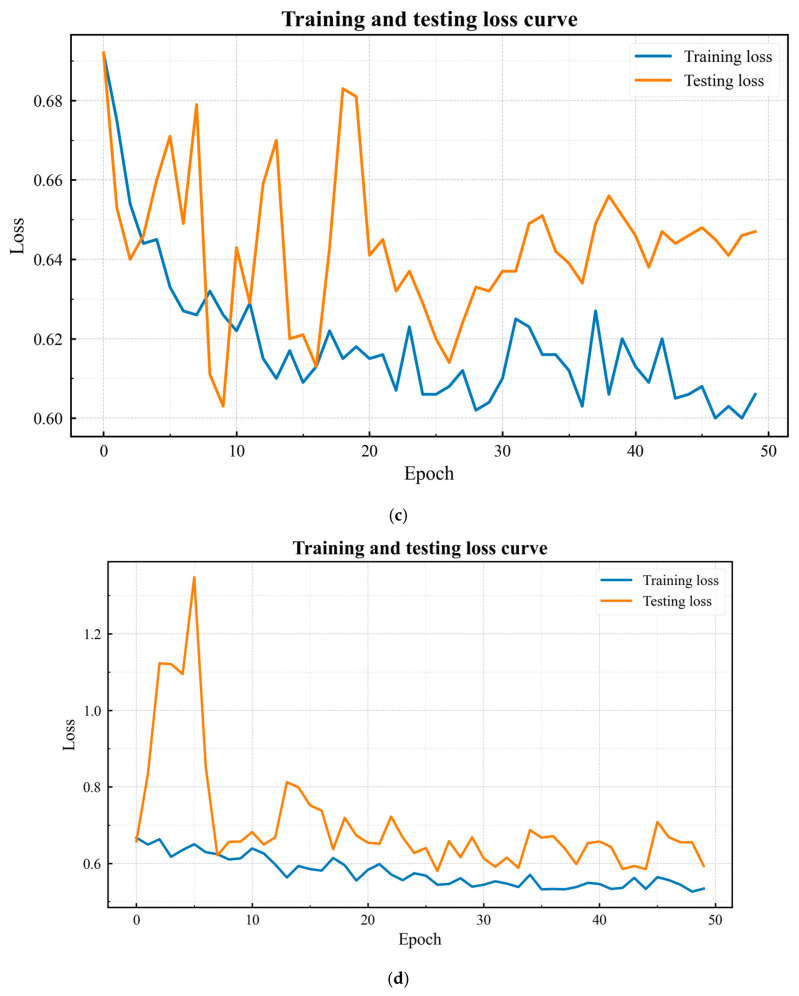
Training results. (**a**) Training accuracy curve, improved PointMLP; (**b**) training accuracy curve, PointMLP; (**c**) training loss curve, top, and improved PointMLP; and (**d**) training loss curve, PointMLP.

The experimental results demonstrate that the improved attention-based PointMLP network exhibits significantly enhanced learning efficiency compared to the baseline model. As shown in Figure 9, the accuracy of the curve for our proposed network improves more rapidly during the initial training phase, achieving competitive performance within fewer epochs. Simultaneously, the loss curve shows a steeper descent, indicating that the network can effectively minimize the objective function and extract discriminative features more efficiently. This accelerated convergence behavior suggests that the introduced attention mechanisms and architectural improvements enable the model to capture more informative point cloud features with greater parameter efficiency.

The results of different algorithms are shown in Table 1. After approximately 50 epochs of training, both networks eventually reach a comparable accuracy of around 90%, indicating that they converge to similar final performance levels. However, the key advantage of our improved architecture lies in its faster convergence speed, which reduces training time and computational costs while maintaining competitive accuracy. These advantages are particularly valuable in practical applications where rapid model deployment and frequent retraining are required. The convergence of both models in later stages suggests that while the enhanced network learns more efficiently, the fundamental representational capacity of the two architectures may be similar at equilibrium. These findings highlight the importance of optimizing not only the final accuracy but also the training dynamics in 3D point cloud recognition tasks. Both PointNet and PointNet++ were tested on their own datasets, with an accuracy of no more than 55%.

**Figure 10:**
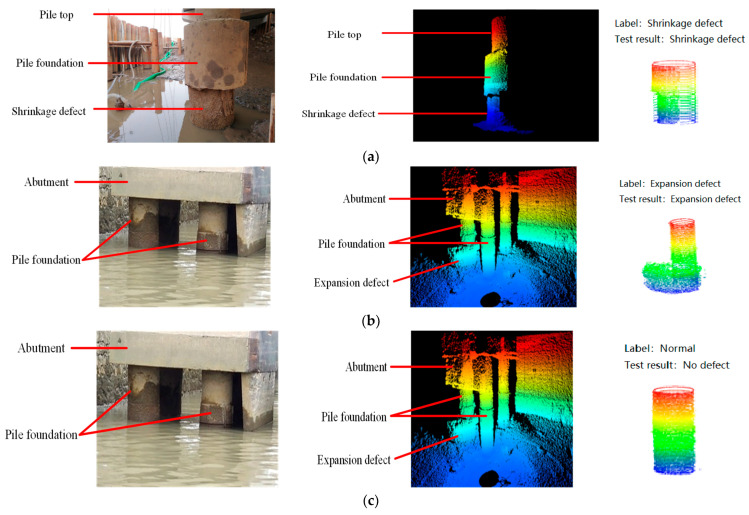
Results of pile foundation defect detection. (**a**) Shrinkage defect detection effect; (**b**) expansion defect detection effect; and (**c**) normal pile foundation detection effect.

The pile foundation single-point cloud dataset obtained, as described in Section 3.3, was applied to the improved attentional PointMLP classification network model for judgment. The results are shown in Figure 10, with an accuracy of 95%. Therefore, the effectiveness and accuracy of the proposed method for underwater pile defect detection based on the diffusion probability model and improved PointMLP are proven. The method uses a 3090 graphics card for computation, with a single frame point cloud processing time of about 50 ms, meeting real-time requirements.

### 4.5. Volumetric Calculation of Pile Foundation Damage

**Figure 11:**
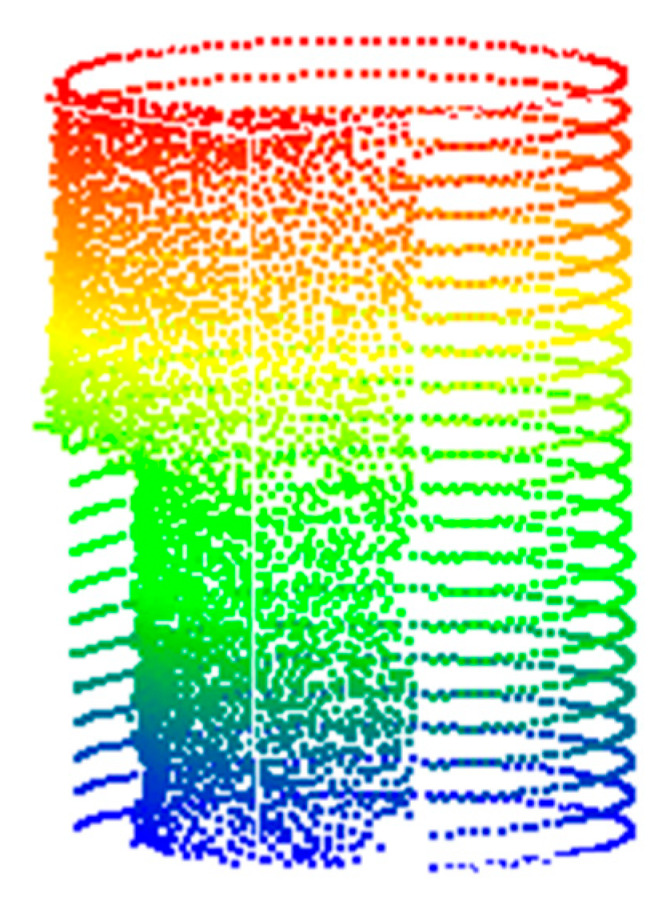
Pile damage calculation results.

In addition to the volume, the algorithm proposed in this paper also calculates the diameter, area, and distance to the mud face in the defect area. The shrinkage of the defective pile foundation in Section 4.5 was tested, and the results are shown in Figure 11. Using the slicing method proposed in this paper, the maximum diameter of the pile foundation was calculated to be 1.5978 m; the minimum diameter was 1.3167 m; the distance from the defective part to the mud surface was 0.2 m; the area of the defective part was 3.4828 m^2^; and the volume of the defective part was 0.4906 m^3^. This algorithm provides a detailed overview of the defective part of the pile foundation and, thus, facilitates a more comprehensive and accurate assessment of the defect.

## 5. Contributions

(1) An underwater pile foundation defect detection method based on the diffusion probability model and improved PointMLP is proposed, as well as a slice method to calculate the pile defect volume. The validity and accuracy of the proposed method were verified by judging and calculating the preprocessed point cloud input model.
(2) To address the geometric irregularity of pile foundation defects, this paper proposes a slicing-based volumetric calculation method for point cloud data, with an error of 0.0756 m^3^.
(3) The experimental results show that the identification accuracy of pile foundation defects is as high as 95%. This method can provide guidance for defect detection in practical engineering applications.

## 6. Conclusions

A novel method based on the diffusion probability model and improved PointMLP is proposed to detect damage in underwater pile foundations. PCA-ICP registration was performed on point cloud data from different stations using sonar systems. Filtering algorithms and Random Sample Consensus (RANSAC) were used to obtain a complete pile foundation point cloud. Pile foundation defect point cloud data were generated and enhanced based on the diffusion probability model. A feature attention mechanism was employed to improve PointMLP, which was trained to identify defects in pile foundations. The actual point cloud data of the dock pile foundation was collected. The experimental results show that the proposed method can effectively identify damage to the pile foundation.

## Figures and Tables

**Table 1:** The results of different algorithms.

Name	Training Accuracy	Test Accuracy
PointNet	94.87%	50%
PointNet++	95.31%	55%
PointMLP	96.87%	85.714%
PoinMLP-SSE (SiLU+Saccuracy of E)	96.25%	95.238%
PoinMLP-RSE (ReLU+SE)	92.75%	90.25%

## Data Availability

The raw data supporting the conclusions of this article will be made available by the authors on request.
